# How Do Mindfulness‐Based and Cognitive Behavioral Therapies Help Adults With Opioid‐Treated Chronic Low Back Pain? A Qualitative Study

**DOI:** 10.1111/papr.70209

**Published:** 2026-10-09

**Authors:** Douwné L. Müller, Eric L. Garland, Robert R. Edwards, Bruce Barrett, Cindy A. Burzinski, Kenneth D. Croes, Robert M. Cradock, Chan Shen, Shilagh A. Mirgain, Janice M. Singles, Aleksandra E. Zgierska

**Affiliations:** ^1^ Department of Family and Community Medicine Penn State College of Medicine Hershey Pennsylvania USA; ^2^ Sanford Institute for Empathy and Compassion University of California San Diego La Jolla California USA; ^3^ School of Medicine, Department of Psychiatry University of California San Diego La Jolla California USA; ^4^ Department of Anesthesiology, Perioperative and Pain Medicine Harvard Medical School, Brigham and Women's Hospital Chestnut Hill Massachusetts USA; ^5^ Department of Psychiatry Harvard Medical School, Brigham and Women's Hospital Chestnut Hill Massachusetts USA; ^6^ School of Medicine and Public Health, Department of Family Medicine and Community Health University of Wisconsin‐Madison Madison Wisconsin USA; ^7^ University of Wisconsin Survey Center Madison Wisconsin USA; ^8^ Department of Surgery Penn State College of Medicine Hershey Pennsylvania USA; ^9^ Department of Public Health Sciences Penn State College of Medicine Hershey Pennsylvania USA; ^10^ School of Medicine and Public Health, Department of Orthopedics and Rehabilitation University of Wisconsin‐Madison Madison Wisconsin USA; ^11^ Department of Anesthesiology and Perioperative Medicine Penn State College of Medicine Hershey Pennsylvania USA

**Keywords:** chronic low back pain, cognitive behavioral therapy, mindfulness‐based therapy, opioid‐treatment, pain management

## Abstract

**Background:**

Chronic low back pain (CLBP) is common and often refractory to existing treatments, necessitating long‐term opioid therapy, which has substantial risks. Mindfulness‐based (MBT) and cognitive behavioral (CBT) therapies are safe and can be effective for CLBP.

**Aims:**

To better understand how MBT and CBT may help improve opioid‐treated CLBP:

**Methods:**

This qualitative study involved a subgroup of participants with opioid‐treated CLBP who completed a 12‐month randomized clinical trial comparing the effects of MBT versus CBT. Participants who reported improvements at 12 months, compared to baseline, in main outcomes (assessed with validated “quantitative” questionnaires) and experience with the study intervention, and who attended the intervention sessions were invited to in‐depth exit interviews. The interviews achieved thematic saturation. Thematic analysis of transcribed interviews summarized qualitative findings.

**Results:**

Fifty participants (26 MBT, 24 CBT) were interviewed. They were, on average, 58.5 (SD = 10.0) years old. Most were female (60.0%), non‐Hispanic (94.0%), and White (82.0%). At baseline, both groups were, on average, moderately‐to‐severely impacted by CLBP and treated with moderate‐to‐high daily opioid doses; at 12 months, they quantitatively reported improved pain, function and QoL scores and reduced daily opioid dose. In their qualitative responses, these participants corroborated the quantitative findings, reporting reductions in back pain (98.0% of interviewed participants), functional limitations (100.0%), and opioid dose (98.0%), with the majority attributing these changes to the study intervention they had received (98.0%, 94.0%, and 90.0%, respectively). Participants also reported the interventions helped them cope with emotional challenges and life stressors, and opioid‐related adverse effects. Participants did not raise concerns about interventions' safety. Qualitative themes identified intervention‐taught techniques participants most frequently used to manage their symptoms.

**Conclusion:**

MBT and CBT can improve health and QoL and reduce opioid dose in adults with opioid‐treated refractory CLBP, calling for improved access to these safe treatments.

## 1 Introduction

Chronic pain, a leading cause of disability, is associated with decreased function, reduced quality of life (QoL), increased anxiety and depression, and dependence on opioids [1, 2, 3]. In 2023, an estimated 24.3% of U.S. adults were affected by chronic pain, exerting a large economic burden on the US healthcare system [4, 5]. Chronic low back pain (CLBP) is the most frequent chronic non‐cancer pain condition among US adults, with a point prevalence of 13.1% in adults 20–69 years old in 2009–2010 [6]. The ongoing prevalence of CLBP highlights the shortcomings of current treatment options [3, 7]. Although various treatments exist, chronic pain is often refractory and many resort to long‐term opioid therapy [1, 3]. However, opioid therapy has been associated with dose‐dependent harms and may not be effective [8, 9, 10, 11]. A recent study found that among adults with CLBP, opioid treatment for up to 12 months was not more effective in reducing pain or disability than non‐opioid treatments [9].

Treatment guidelines recommend nonopioid therapies as the first line treatments for chronic pain [9, 12, 13]. Mindfulness‐based (MBT) and cognitive behavioral (CBT) therapies have shown efficacy in improving outcomes in chronic pain, including CLBP [2, 12, 14, 15], including in recent randomized clinical trials (RCTs) [16]. CBT is one of the most widely used psychological interventions in chronic pain care, seeking to change how individuals experience pain [17, 18]. It often includes techniques intentionally diverting attention from unwelcome physical or emotional experiences by helping individuals modify unhelpful thoughts, emotions, and behaviors while fostering the development of more effective coping skills [18, 19]. Mindfulness promotes a non‐judgmental, non‐reactive awareness of present‐moment experiences, including bodily sensations, thoughts, and emotions, thus encouraging acceptance and reinterpretation of pain as sensory information, a therapeutic process that may contribute to the pain‐relieving effects of mindfulness [15, 20, 21]. Although MBT and CBT are safe, acceptable, and can help treat chronic pain, there is a lack of information on long‐term effects of MBT and CBT on chronic pain, especially in opioid‐treated populations and when comparing treatments [2, 17, 22].

To better understand the potential of MBT and CBT for improving outcomes over the longer‐term in refractory, opioid‐treated CLBP, this qualitative study involved a subgroup of adults with opioid‐treated CLBP who participated in the multisite, pragmatic RCT (*N* = 770), *Strategies To Assist with Management of Pain* (STAMP), that compared the effectiveness of MBT versus CBT over one year [23, 24]. The study participants were randomized to either MBT (*n* = 385) or CBT (*n* = 385) interventions, which comprised eight weekly group therapy sessions, and completed assessments every 3 months, from baseline through 12 months (study exit). Main outcomes (back pain, function, QoL, and opioid dose) were assessed with validated quantitative surveys [23]. In addition to surveys, all participants completed brief qualitative interviews at baseline and exit on their experiences living with opioid‐treated CLBP and with the study participation and interventions, and a subset of participants who had attended the intervention sessions and reported satisfaction with the intervention and improvements across the main outcomes was invited to complete qualitative in‐depth exit interviews.

STAMP survey's “quantitative” results showed that both MBT and CBT groups significantly reduced pain, functional limitations, and opioid dose, and increased mental and physical health‐related QoL post‐intervention through 12 months, without differences between the groups [16]. However, the lack of an “inactive” comparison group (such as “usual care‐only”) precluded drawing firm conclusions about the effectiveness of study interventions versus influences of other factors and regression to the mean effects. Brief qualitative assessments highlighted the heavy toll of CLBP and opioid therapy on participants at baseline, and that the study interventions were viewed as helpful at the study exit [25, 26].

The present study describes findings from the in‐depth exit interviews with this subgroup of STAMP participants (*N* = 50) to help answer questions about what, if anything, including the study interventions, helped these participants improve their function and QoL, reduce pain and opioid medication use, and cope with stressors and negative impacts of opioid‐treated CLBP.

## 2 Methods

### 2.1 Study Design

This qualitative study followed the consolidated criteria for reporting qualitative research (COREQ) checklist [27]. It was part of STAMP, the parent multisite RCT (*N* = 770) that compared the effectiveness of MBT versus CBT over 12 months among adults with opioid‐treated CLBP [23]. Details of the STAMP trial are described elsewhere [23]. The CBT and MBT interventions consisted of eight weekly, therapist‐led, 2‐h group sessions and home practice recommendations. Outcomes assessments took place at baseline, then every 3 months through 12 months (exit) and comprised validated questionnaires. Those who completed exit assessments were considered for the in‐depth exit interviews, which continued from June 2019 through March 2023.

The study was approved by the Institutional Review Boards (IRBs) of the participating institutions, with the University of Wisconsin‐Madison's IRB serving as the IRB of record (study #2017‐0353). Certificate of Confidentiality was issued by the National Institutes of Health. The trial was registered with ClinicalTrials.gov (#NCT03115359) before participant enrollment. The independent Data and Safety Monitoring Committee reviewed study progress and participant safety twice annually. Participants completed written informed consent procedures before enrollment and were compensated for their participation.

### 2.2 Setting and Participants

The STAMP study was completed between July 2017 and November 2022, with participants recruited from clinics and community settings [23]. Participant eligibility criteria were age ≥ 21 years old, English fluency, and CLBP with an “average pain” severity score ≥ 3 on the Brief Pain Inventory (BPI) 0–10 numerical rating scale (with 0 = no pain) [28], a score ≥ 21 on the Oswestry Disability Index (ODI; total score 0–100, with 0 = no back pain‐related function limitations) [29], and daily opioid dose ≥ 15 morphine milligram‐equivalents (MME) for at least 3 months [23].

Among 770 (385 MBT, 385 CBT) STAMP participants, 502 (258 MBT, 244 CBT) completed their 12‐month assessments. To be considered for the in‐depth exit interviews, participants needed to complete their exit assessment, attend at least 4 intervention sessions, and report improvements, compared to baseline, in primary (pain, function) and secondary (health‐related QoL, daily opioid dose) quantitatively‐assessed outcomes, and positive impact of the study on these outcomes. Selection of the in‐depth interview candidates (completed by A.E.Z. and C.A.B.) also strived to balance participation by the study recruitment site (Madison, Boston, Salt Lake City) and intervention arms (CBT, MBT). Research personnel contacted the selected subgroup of eligible STAMP participants to obtain their verbal consent before individual in‐depth interviews were scheduled with the University of Wisconsin Survey Center (UWSC) qualitative researchers. Participants were compensated $50 for completing an in‐depth interview.

### 2.3 Data Collection

**Quantitative data** were collected by self‐report at baseline, 3, 6, 9, and 12 months using validated questionnaires. Co‐primary measures included “average pain” severity (BPI [28]) and CLBP‐related functional limitations (ODI [29]). Secondary outcomes included mental and physical health‐related QoL (Medical Outcomes Study Short Form, 0–100 total score; 0 = worst QoL [30]) and daily opioid dose (Timeline Followback [31]; MME/day, averaged over the “past 14 days” and calculated using established conversion factors and methods [32]). Participant‐perceived change in pain severity, function, the need for opioid medications, overall health, and overall treatment satisfaction were assessed with a Treatment Satisfaction Survey, adapted from the Patient Global Impression of Change (PGIC) [33]. The Treatment Satisfaction Survey's 1–7 ordinal responses were classified into three broader categories: (a) “better” or “improved” status (responses “5–7”); (b) “stable” or “unchanged” status indicating no meaningful change (responses “2–4”); or (c) “worse” status (response “1”). Surveys also collected baseline data on demographics, CLBP‐related characteristics [34], and healthcare utilization and productivity [35]. Intervention session attendance was recorded by research personnel. These data were collected as a part of the main study and used for the present analysis to document that the subset of participants who completed in‐depth interviews was properly selected, per eligibility criteria.

**Qualitative in‐depth exit interviews** collected qualitative information relevant to STAMP's main outcomes and study interventions. The in‐depth interviews followed a scripted guide (Data S1), developed between February and June 2018 by the research team members in collaboration with stakeholder‐partners. The interviews were conducted by phone by the UWSC researchers (R.M.C., K.D.C.), audio‐recorded, and transcribed verbatim. No repeat interviews to elicit additional information were conducted, and no transcripts were returned to participants for feedback. A saturation analysis [36], completed after 50 interviews, established that thematic saturation was reached.

The interview focused on six main categories relevant to opioid‐treated CLBP and aligned with the STAMP study's goals to assess if the MBT/CBT interventions could help participants improve their outcomes (pain, function, opioid use, and psychological distress related to their condition; Categories 1–4), help cope with opioid‐related side effects (Category 5), and whether the interventions were associated with any negative effects (Category 6).

The interview questions first asked about the overall perceived change (Yes/No) in pain (Category 1), function (Category 2) and opioid use (Category 3) (Data S1, Questions 202, 302, 402). Within each category, several additional questions were asked: two inquired about what helped in general (“What has helped you [achieve these improvements]?”) and which of these strategies helped most (Data S1, Questions 208, 309, 408), with open‐ended responses. Participants were also asked (Yes/No) if the interventions helped them improve outcomes (Data S1, Questions 206, 307, 406) and, if yes, how they helped achieve these improvements.

Participants were also asked about the presence (Yes/No) of mood, stress, or anxiety about their back pain, daily activities, or opioid medications (Category 4) (Data S1, Questions 209, 310, 409). This question was followed by inquiry whether the study intervention helped reduce this emotional distress (Data S1, Questions 210, 311, 410) and how the interventions helped participants cope with it (Data S1, Questions 211, 312, 411).

A question about ever experiencing adverse effects of opioids (Category 5) was also posed; those who endorsed opioid‐related adverse effects were asked to describe them, whether the study interventions helped them cope with these adverse effects, and if yes, how they helped (Data S1, Questions 501–504).

Finally, two questions explored the safety of the study interventions (Category 6) and asked about any negative effects associated with the MBT/CBT practice and, if yes, to describe them (Data S1, Questions 505, 506).

### 2.4 Data Analysis

**Quantitative data** were analyzed using R statistical software (Version 4.3.3, R Core Team 2023). Descriptive statistics (mean ± standard deviation or frequencies) described the study sample. Within‐group change in outcomes at 12 months, compared to baseline, was assessed with a paired‐samples *t*‐test, with a statistical significance level set at two‐tailed *p* < 0.05. To normalize the distribution and reduce the influence of extreme values, a logarithmic transformation was applied to the opioid dose data.

**Qualitative data** were analyzed using the applied thematic analysis approach [37] and NVivo software (Release 1.7). Themes were conceptualized prior to coding by reading through intervention manuals and interview transcripts of the first few interviews. To operationalize themes, a mix of deductive (derived from the intervention manuals) and inductive (from respondents' responses) codes were developed. Examples of deductive codes include: “Learned to do physical activity or keep active” and “Learned to be aware of automatic thoughts or beliefs or reactions.” Inductive codes emerged from participants' experiences, such as problems related to using opioid medications, and included codes such as: “Addiction, craving or withdrawal”, “Constipation or digestive issues,” and “Impaired cognition.” Validity of the coding process was enhanced in a few ways. First, the study research and stakeholder‐partner teams reviewed and provided feedback on the codebook. Second, transcripts were coded by two analysts (R.M.C., K.D.C.), with one serving as the initial coder for approximately half of the transcripts, and another reviewing the coding, then reversing roles for the remaining transcripts. The reviewer‐coder could either agree or disagree with the initially assigned codes and apply their own codes, which were then reviewed by the initial coder. Coders used a consensus approach, meeting frequently to discuss and resolve differences in code applications and refining code definitions in the codebook as needed to clarify code application. Third, prior to its finalization, the coding results were reviewed by the researchers (A.E.Z., R.R.E., E.L.G.) who also provided input in case of disagreements between the primary coders.

## 3 Results

### 3.1 Baseline Characteristics

A subgroup of 50 STAMP participants (26 MBT, 24 CBT) were interviewed for this study. They were, on average, 58.5 ± 10.0 years old, with most being female (60.0%), non‐Hispanic (90.0%), and white (82.0%) (Table 1).

**TABLE 1:** Baseline characteristics of the study sample (*N* = 50).

Variables	Total sample *N* = 50	MBT group *N* = 26	CBT group *N* = 24
Demographic characteristics			
Gender, *n* (%):			
Female	30 (60.0%)	18 (69.2%)	12 (50.0%)
Male	20 (40.0%)	8 (30.8%)	12 (50.0%)
Age, years, mean (SD)	58.5 (10.0)	59.6 (9.2)	57.4 (11.0)
Ethnicity *n* (%):			
Hispanic or Latino	2 (4.0%)	0 (0.0%)	2 (8.3%)
Non‐Hispanic	47 (94.0%)	25 (96.2%)	22 (91.7%)
Race *n* (%):			
Black	5 (10.0%)	3 (11.5%)	2 (8.3%)
White	41 (82.0%)	23 (88.5%)	18 (75.0%)
Other	4 (8.0%)	0 (0.0%)	4 (16.7%)
Primary and secondary outcomes			
Average pain severity (BPI, 0–10 score), mean (SD)	5.8 (1.6)	5.4 (1.5)	6.1 (1.5)
Functional limitations (ODI, 0–100 score), mean (SD)	47.1 (13.7)	46.9 (13.7)	47.4 (13.9)
Physical health‐related QoL (SF‐12, 0–100 score), mean (SD)	28.3 (8.6)	28.2 (8.6)	28.3 (8.8)
Mental health‐related QoL (SF‐12, 0–100 score), mean (SD)	41.7 (10.8)	41.1 (10.9)	42.4 (10.8)
Opioid dose (TLFB)			
MME/day, mean (SD)	112.0 (261.0)	65.2 (88.9)	163.0 (363.0)
Log MME/day, mean (SD)	5.4 (1.7)	5.3 (1.3)	5.5 (2.0)

*Note:* The frequencies/percentages in a given variable category may not total the sample size per group or 100.0% responses as some participants' responses were unavailable (e.g., missing or marked as declined, unknown or unspecified).

Abbreviations: BPI, brief pain inventory; CBT, cognitive behavioral therapy; MBT, mindfulness‐based therapy; ODI, Oswestry disability index; QoL, quality of life; SD, standard deviation; SF‐12, 12‐item short form health survey; TLFB, timeline followback.

As presented in Table S1, the majority of participants experienced at baseline long‐standing CLBP, tried many prior treatment options, and were substantially affected by their CLBP, with many not working and reporting negative impact of CLBP on their productivity at work or home as well as heavy utilization of healthcare resources. Participants indicated experiencing, on average, 59.0% ± 21.2% of pain relief from their opioid therapy.

At baseline (Table 1), the sample's mean scores of the BPI's “average pain” severity (5.6 ± 1.8) and ODI's CLBP‐related functional limitations (47.1 ± 13.7) were in the moderate range. Their SF‐12's mean scores were below the average U.S. population mean standardized score (set at 50 points) [30, 41], indicating compromised physical and mental health‐related QoL (Table 1). The mean opioid dose was high (112.0 ± 261.0 MME/day), with substantial variability within the sample.

### 3.2 Change in Quantitative Outcomes at 12 Months

Consistent with the eligibility criteria for the in‐depth exit interviews, all 50 participants attended at least 4 intervention sessions, with 16.0% attending 4–6 sessions and 84.0% attending 7–8 sessions. They also reported, across both MBT and CBT groups, significant reductions in the pain severity and functional limitation scores, log‐transformed daily opioid dose, and increases in the physical and mental health‐related QoL scores at 12 months, compared to baseline (Table S2). Most participants in both groups also reported perceived improvements in their pain, function, opioid needs, overall health, and satisfaction with the received intervention (Table S2).

### 3.3 Qualitative Findings

On average, the qualitative interviews took 35 min (range: 12–76 min). What follows is the summary of qualitative analysis results across six a priori defined categories. Details of identified themes within each category, along with representative quotes on the intervention effects, are presented in Table 2.

**TABLE 2:** Qualitative responses: participants reporting improvements in specific domains relevant to and the frequency of study intervention‐related techniques noted as being “most helpful” for managing symptoms in opioid‐treated CLBP.

Variable	Participant responses
Back pain	
Reduced Pain	98.0% (25 MBT, 24 CBT participants)
Improvement linked to study intervention	98.0% (25 MBT, 24 CBT participants)
Study intervention‐based technique reported as the most helpful strategy	MBT: 61.5% (16 MBT techniques of 26 “most helpful” strategies) CBT: 77.3% (17 CBT techniques of 22 “most helpful” strategies)
Function (Daily activities)	
Improved function	100.0% (26 MBT, 24 CBT participants)
Improvement linked to study intervention	94.0% (24 MBT, 23 CBT participants)
Study intervention‐based technique reported as the most helpful strategy	MBT: 77.8% (21 MBT techniques of 27 “most helpful” strategies) CBT: 88.0% (20 CBT techniques of 25 “most helpful” strategies)
Opioid medication use	
Reduced daily opioid use	98.0% (25 MBT, 24 CBT participants)
Improvement linked to study intervention	90.0% (24 MBT, 21 CBT participants)
Study intervention‐based technique reported as the most helpful strategy	MBT: 66.7% (16 MBT techniques of 24 “most helpful” strategies) CBT: 78.3% (18 CBT techniques of 23 “most helpful” strategies)
Mood, stress or anxiety related to opioid‐treated CLBP	
Back pain	
Presence of emotional distress	86.0% (23 MBT, 20 CBT participants)
Interventions helped cope with distress	95.3% (21 MBT, 20 CBT participants)
Function (Daily activities)	
Presence of emotional distress	80.0% (23 MBT, 17 CBT participants)
Interventions helped cope with distress	94.7% (20 MBT, 16 CBT participants)^a^
Opioid medications	
Presence of emotional distress	50.0% (15 MBT, 10 CBT participants)
Interventions helped cope with distress	78.3% (10 MBT, 10 CBT participants)^a^
Side effects of opioids	
Presence of side effects	68.0% (18 MBT, 16 CBT participants)
Interventions helped with side effects	58.8% (13 MBT, 7 CBT participants)

Abbreviations: CBT, cognitive behavioral therapy; CLBP, chronic low back pain; MBT, mindfulness‐based therapy.

^a^ This question was inadvertently omitted for 2 participants who endorsed distress.

#### 3.3.1 Back Pain

Reduction in back pain was reported by 98.0% of the interviewed participants, with 98.0% linking it to the study intervention (Table 2). Non‐intervention‐related strategies mentioned as useful for back pain management included “medications,” “ice or heat,” and “resting or lying down.”

When asked about what helped them most in reducing back pain over the past 12 months, whether intervention‐related or not, CBT participants identified 22 and MBT participants identified 26 different “most helpful” strategies, with the majority of these strategies coming from the study interventions (Table 2). Qualitative responses to the question of how the interventions helped reduce back pain revealed several key themes of most frequent intervention‐specific strategies: “learning to do physical activity or keeping active”, “learning relaxation, calming or stress reduction techniques”, “learning to practice meditation or present‐moment awareness” and “breathing techniques” (Table 3).

**TABLE 3:** How the study interventions helped participants (*N* = 50) reduce symptoms of chronic low back pain and daily opioid dose: Themes and representative quotes.

Themes	Example quotes
**How has MBT or CBT helped you reduce your back pain?**	
Learned to do physical activity or keep active	“I learned a little bit of techniques from the class on how to do exercise and how to control the pain.” (CBT participant) “Movement, additional movement of any sort, be it exercise at home, be it walking, any kind of movement just to keep it loose and limber has helped a lot.” (CBT participant) “Working with the legs, too. All of these were like activities that we did physically, you know, with the leg and…the arms, and it helped, while we were there in the class.” (CBT participant) “I think it makes my back stronger by being active, working all those back muscles and everything. You know, having the same goal that I'll swim every other day for 20 laps, you know, making goals like that.” (CBT participant) “Exercise, light exercise. Don't be afraid of—it will initiate or trigger pain. But, you know, you do exercise.” (CBT participant) “Getting up and moving more.” (CBT participant) “I do my stretches, you know. I try to keep my back aligned to where I wouldn't have as much trouble with the nerve, because this nerve pain, that's probably the worst pain I've had.” (MBT participant) “I also started doing some stretching. I found that, because I was being more mindful, I was not just staying in one position for a long period of time. I was, you know, doing stretches. I was incorporating some light exercise into different things that I did during the day.” (MBT participant) “I spend 15 min, twice a week on the treadmill and when I'm on the treadmill, I will play very relaxing music, and close my eyes, and I meditate on that, and that has been a big help.” (MBT participant)
Learned relaxation, calming or stress reduction techniques	“For me, some of the relaxation techniques work the best…Going through, kind of, the different muscle groups and mindfully controlling them to relax.” (CBT participant) “I learned—she called it a progressive muscle relaxation, and that has been very helpful to me when I'm having a real good pain episode. And when I go to sleep at night, I remember how she guided us through it. And I did that for myself, and I find it to be really, really helpful.” (CBT participant) “I incorporated a relaxation element. That being, trying to go somewhere in nature, you know, to a place that's soothing and calming, you know, for me. For some people that's the middle of the city, doesn't matter, but like whatever feels good. It's important, I think, to just incorporate the relaxation part into the pain management part. I think those two go hand in hand…because of the anxiety producing factor of pain, and then once you have the anxiety, that it maybe could produce even more pain. So if you can, like, alleviate anxiety…through relaxation, I think that that can ultimately help pain.” (CBT participant) “They taught us how, you know, using the progressive muscle relaxation, I guess we call them PMRs, and that helped so much.” (CBT participant) “I'm really going to say the CBT and the PMRs”. (CBT participant) “I do meditate, and I do a lot of thoughts of visualizations of my body relaxing and healing.” (MBT participant) “It's helped me deal with the pain. And when I do the breathing, it makes me relax.” (MBT participant) “Once I took the STAMP class, I learned more how to relax and how to imagine and how to, you know, handle the things—I mean, not just the pain but just life in general.” (MBT participant) “While doing the meditation, I would be relaxed. It would make me more relaxed. And some of the issues I had was with my back. Well, the main issue I had was with my back, and I guess the sciatic nerve issue…And the being relaxed like that seemed to take the pressure off of the sciatic nerve.” (MBT participant) “I believe it has helped me because it has given me a chance to slow down and relax my entire body and release all of my stress. I believe that a large part of my back pain is from—I believe that's where I do carry most of my stress at.” (MBT participant)
Learned to practice meditation or present‐moment awareness	“Kind of meditate and, you know, focus on, you know, what the pain feels like in a certain area.” (CBT participant) “Just be still and focus on my pain and work on reducing the, how should I say, reducing the area of the pain to where the pain is—to save it from truly becoming a problem, instead of just making it be, having it be like overall pain.” (CBT participant) “One of the things I gleaned out of that was being able to pay closer attention to when this pain started to come on so that I could take actions, like meditation…so that I could have a little better control over the amount of pain I felt.” (MBT participant) “The ability to like visualize the pain shrinking and visualize like the pain as a color, and while I'm trying to do my mindfulness now, I try to change that color. And I believe that's helped.” (MBT participant) “Usually, like when I'm at work and I don't want to take any kind of medication, I will use some of the mindfulness techniques to visualize. One of the things that they had taught me was you visualize like a river and your pain going down with it. So that has helped me.” (MBT participant) “Without a doubt it's been the mindful mediation, listening to the CDs.” (MBT participant) “I think the mindfulness. And that is because when I do have like a flare up, it doesn't really matter where I'm at or what I'm doing. You know, I can always take five minutes and escape to the restroom to try to help that pain to dissipate, whereas, you know, if I'm in the company of others or some place, it's hard to lay down and do the back exercises.” (MBT participant) “That mindfulness training really made a difference and helped me.” (MBT participant) “The mindfulness, I would say, is the number one thing that helped the most.” (MBT participant)
Breathing techniques	“I think the breathing, yeah, learned in the cognitive behavioral therapy. I can use that during the day…It doesn't have to be just pain, it can be stressful, or mental, or emotional. Just that slowing down and stopping and breathing and, yeah, finding release through that breathing exercise…It's made more change than anything.” (CBT participant) “The breathing was the main thing for me.” (MBT participant)
**How has MBT or CBT helped you improve your ability to do your daily activities?**	
Learned to do physical activity or keep active	“It made riding my bicycle easier to do because talking about pain gates and opening and closing the gate, it's about finding an exercise that, you know, gives us what makes us feel good, not necessarily a full endorphin, like a marathon runner, but exercise to go out and do stuff and say hi to neighbors by controlling those pain gates.” (CBT participant) “Just keeping moving, making sure that, you know, you're aware of keeping yourself on the move and going.” (CBT participant) “Do a little bit exercise every day, and keep the muscle stretched.” (CBT participant) “Movement is number one.” (CBT participant) “Doing exercises.” (MBT participant) “Stretching and just being active actually does improve my ability to be active, if that makes sense…Even if I just decide to go out for a walk, sometimes I just have to push beyond the first, you know, block or two because I feel like I don't really want to go for a walk because my back is sore, or whatever, but once I do it, it's better. It seems to get better the farther I go.” (MBT participant) “Stretching and just trying to get outside and do a little bit of walking, even though that might not be what I feel like, but just getting outside of myself and doing something.” (MBT participant)
Learned relaxation, calming or stress reduction techniques	“The PMR, the one that you sit in the chair. I do that often. And that helps a lot. It helps bring my leg up and hold it real— there was three of them, the leg, the arm, and the back… And they said if you just do it constantly, it'll get better. And I think it does. It really does.” (CBT participant) “By practicing the breathing relaxation exercise right before I even go to the golf course, or go out in the yard to plant, or weed, or whatever I'm doing around the house. Knowing that if I take that moment, that 15–20 min, it's going to be much easier and, yeah, less painful, better.” (CBT participant) “I think it's that one where I focus on the body and just try to relax. I think that one I do more often because that one seems to help the most.” (CBT participant) “The CBT training is right up there because of the fact that my back is— I don't have to take opioids anymore, which is like a miracle kind of thing, you know, and that, yes, it's very not often. I might get a little bit of pain, but nothing like I had before and I know what to do. I'll, you know, go back to my PMRs. I'll do those. You know, and it's never gotten to the point where I've had to take Dilaudid again, so that to me, is great.” (CBT participant) “It keeps my mind calm and clear, and—just calm.” (MBT participant) “I would say the breathing. Learning how to breathe and to relax and not stress over, I guess, the pain so much.” (MBT participant)
Learned to practice meditation or present‐moment awareness	“I can go into, you know, a mindfulness situation while I'm doing something, just go slower, and easier, more profound, and that's been helping out a lot.” (MBT participant) “I would say that there are times when I'm maybe having an especially painful day where I'll take time out of the day, and lay down, and do like a full body scan. That's one of the meditation things we did, and it just kind of resets my whole self and then I'm able to go back to whatever I was trying to do, or whatever.” (MBT participant) “I go into the way they were showing us how to do the meditation, you know, how to listen to things and how to pay more attention to things.” (MBT participant) “Without the mindfulness training, I wouldn't have done the other things. So I guess I would have to say the mindfulness training.” (MBT participant) “Most helpful is definitely the mindfulness training, the breathing.” (MBT participant) “The mindfulness training.” (MBT participant)
Learned pacing or setting realistic expectations or limits	“Just setting a pace, setting a slower pace, you know, realizing that, not internalizing everything that I'm doing as, you're a failure because you can't do it. Just realizing, hey, you did this much. That's pretty damn good.” (CBT participant) “I make it realistic, because I wasn't realistic before.” (CBT participant) “The big thing is I don't expect so much out of myself like I used to.” (MBT participant)
**How has MBT or CBT helped you reduce your use of opioid medications for your back pain?**	
Learned to do physical activity or keep active	“Staying active…The importance of movement”. (CBT participant) “Movement, mobility.” (CBT participant) “Movement. Moving my body, being in a place where I'm not sedentary a lot of the time, being mindful of like how much exercise I'm getting.” (CBT participant) “Staying active, walking. (I: And here, can you tell me a little bit more about what staying active means for you?) Just building more of my back muscles and, you know, the rest of my muscles that I had before all this started, a lot of it had atrophied because I couldn't move around after my surgery.” (CBT participant) “Stretch a bit, do something that'll maybe help and not have to take the medication…Being active does help”. (MBT participant) “I would say a piece of the puzzle would be the back exercises, just walking more, getting physical exercise for my back.” (MBT participant)
Learned relaxation, calming or stress reduction techniques	“It has taught me not to be so stressed out about having pain. Using the cognitive behavioral therapy, incorporated with the meditation, has taught me to release like, stress. And not be so tense.” (CBT participant) “A couple of back‐to‐back PMR exercises.” (CBT participant) “The relaxing, the mindfulness, it all just—because you know it's like—I got to say it because, it's like when the pains come in, oh, oh, let me see, like, oh my God, you take—you know, you're trying to be ahead of the pain and stuff, so try to get your mind off of it, like relax.” (CBT participant) “The single most helpful thing, if I have to pick one, it's the PMR.” (CBT participant) “The ability to stop and calm yourself down and calm yourself down and slow your breathing.” (CBT participant) “Stress reduction, you know, so, instead feeling so anxious that you want to medicate it away, you just give yourself some time, you know.” (MBT participant) “When the pain starts hurting, I start breathing, and I am able to breathe and relax, or sometimes I'll just take a complete break for 5 min, sit down, and breathe. And then I'm not stressed over it, and I can delay taking pain pills, and so I'm not taking them as often.” (MBT participant) “I just feel like, because I'm able to do that, and physically— well, release emotionally and physically, that I am releasing that stress and tension in my back. And that, therefore, you know, makes the pain go away a bit. And so I believe that's how it's helped me reduce opioids.” (MBT participant)
Learned to practice meditation or present‐moment awareness	“Mindful meditation puts me in the moment. Instead of letting my mind wander and get fixated on the pain, mindful meditation kind of snaps me out of that mode, or thinking…” (MBT participant) “Oh, definitely the mindfulness. Because I will think about—I will be okay if I just sit and relax and think about something else, and then my back pain is relieved some.” (MBT participant) “A lot of it had to do with mindfulness and the, you know, exercises that I did, the meditation exercises.” (MBT participant) “The meditation, you know, being able to focus on my pain and be able to relieve pain on my own through what I've been taught.” (MBT participant) “The mindfulness training.” (MBT participant) “I think the meditation was the big thing.” (MBT participant)
Learned to be aware of automatic thoughts or beliefs or reactions	It's kept me from catastrophizing, like, I can't make it without the medication, to, I don't need this medication. My body's doing well. It's an interesting problem, but I can overcome it. I don't need all these medications. (MBT participant) It kind of makes me think before I just pop a pill. Yeah, and it's made me kind of think first. (MBT participant) Before I would take it [opioid medication], I would go into what she had taught us about, going into a place and just kind of meditating a little bit, and asking, why am I taking this? What am I taking? I can't remember all the questions either, but just be more aware and mindful of what I'm doing, and that would often delay needing to take that. (MBT participant) “I just basically don't reach for the bottle…I consider all of the alternatives first and exercise some of the alternatives and then make a decision.” (MBT participant)
**How has MBT or CBT helped you improve your mood, stress, or anxiety about your back pain, daily activities, or opioid medications?**	
Learned relaxation, calming or stress reduction techniques	“I'm more focused with my progressive muscle relaxation, making sure that I'm doing everything I'm supposed to do to be relaxed, to be focused, to be conscious in the moment, not worrying about tomorrow, not worrying about next week.” (CBT participant) “When the pain come I try to relax because I get filled up with anxiety and stuff, because I be trying to think about how long is it going to stay, and trying to be ahead of it.” (CBT participant) “I think it's just helped me be a better person because like instead of being snappy when I don't feel good, I'm able to be more calm and address things. So I think it's helped with relationships as well.” (MBT participant) “I just basically don't reach for the bottle…I consider all of the alternatives first and exercise some of the alternatives and then make a decision.” (MBT participant)
Learned to be aware of automatic thoughts or beliefs or reactions	“It gives me a different perspective than I once had about it. I catastrophized about it a lot before and, you know, excused myself for being grumpy or whatever. My mood's better. I just think it's changed my life.” (MBT participant) “It puts me in a different perspective of not being down on myself because I can't do anything. But doing the mindfulness allows me to kind of be in a different head space where I feel more centered…” (MBT participant) “I used little snippets of mindfulness a lot just in terms of any time I got anxious about anything. And that included medications. And it was more of a check in where I'm afraid maybe it'll be too soon to take my medication now, or, do I really need a pill? And sometimes if I checked in with the mindfulness, I could check in with my pain, and be able to realize that, Okay, if you let yourself relax, if you let that anxiety go, you can feel where your pain is, and reevaluate whether I did or really didn't need a pain med.” (MBT participant) The anxiety that I used to have with things, with pain, How am I going to do this? How am I going to do that? How am I going to do something else? You know?…I just find myself now not being in that same, you know, frame of mind, or physical angst, or whatever. (MBT participant) “It lets me pause and seek other options before becoming panicked and running to the opioids.” (MBT participant)
**How has MBT or CBT helped you cope with opioid‐related side effects or other problems?**	
Learned to be aware of automatic thoughts or beliefs or reactions	[Reported side effects of addiction, constipation, and euphoria] “I carried the pill bottle in my pocket everywhere I went. I mean, it was a go‐to. And once I started to stop, to breathe, to think, then the outcome was different. I didn't take as much or not at all.” (MBT participant) [Reported side effect of being less active] “The biggest thing I can say is that what the number one benefit for me was, was the way I looked at things. Again, that you've got to be, you know, for me, I have to be positive. Look at, I don't know, like your mom used to say, the brighter side of things.” (MBT participant)
Learned to practice meditation or present‐moment awareness	[Reported side effect of being less active] “When I'm more in mindful meditation, and the music, and those […] tools that I can use, I don't find myself sitting on the couch just vegetating. I don't have time for that anymore and I don't do it.” (MBT participant) [Reported side effects of disorientation or nausea and impaired cognition] “[When] I took a pill, and it started to make me feel nauseous, and I did have to lie down, […] I did do, you know, headphones and mindfulness to try to get through that.” (MBT participant)
Training motivated me to reduce opioid medications	[Reported side effects of constipation or digestive issues and impaired cognition] It coaxed me into reducing my pain medication. (CBT participant) [Reported side effect of drowsiness or fatigue] I would sit and take the time for myself and think I'm getting all these side things that are happening. I'm getting too sleepy, I'm getting non‐functioning, I'm getting, you know, and think, I can do this thing. I've got to stop this, you know, depending on the pain medicine. I've got to depend on something else […]. And that would be the mindfulness, doing things for myself. (MBT participant)

Abbreviations: CBT, cognitive behavioral therapy; MBT, mindfulness‐based therapy; PMR, progressive muscle relaxation.

#### 3.3.2 Function (Daily Activities)

Improvements in daily activities were reported by all participants, with 94.0% reporting the study interventions helped them achieve it (Table 2). Participants also mentioned non‐intervention‐related helpful strategies such as: “medication,” “being aware of what causes pain or finding alternatives,” “ice or heat,” and “massage.”

When asked what most helped them improve their function over the past 12 months, whether intervention‐related or not, CBT participants identified 25 and MBT participants identified 27 “most helpful” strategies, with the vast majority of them stemming from the study interventions (Table 2). Qualitative responses to the question of how the interventions helped improve daily activities revealed several key themes, with the most frequent being: “learning to do physical activity or keeping active”, “learning relaxation, calming or stress reduction techniques”, “learning to practice meditation or present‐moment awareness”, and “learning pacing or setting realistic expectations or limits” (Table 3).

#### 3.3.3 Opioid Medication Use

Reductions in opioid use were reported by 98.0% of participants, with 90.0% of them attributing this change to the study intervention (Table 2). Participants also mentioned non‐intervention‐related strategies to reduce opioid use, such as: “controlling how or how much or when pain medications are taken,” “ice or heat,” “being motivated to reduce pain medications,” and “eating right or maintaining the right weight.”

When asked about the most helpful strategies for reducing their opioid medication use, whether intervention‐related or not, CBT participants identified 23 and MBT participants identified 24 different strategies. The majority of the “most helpful” strategies came from the study interventions (Table 2). Qualitative responses to the question of how the interventions helped reduce opioid use revealed several themes, which included: “learning to do physical activity or keeping active,” “learning relaxation, calming or stress reduction techniques,” “learning to practice meditation or present‐moment awareness,” and “learning to be aware of automatic thoughts or beliefs or reactions” (Table 3).

### 3.4 Mood, Stress or Anxiety Related to Opioid‐Treated CLBP

A substantial proportion of participants indicated presence of mood, stress, or anxiety about their back pain (86.0%), daily activities (80.0%), and opioid medications (50.0%); the majority reported the interventions helped them improve coping with these emotional challenges (Table 2).

When asked how the interventions helped with these emotional challenges, the most frequent themes included “learning relaxation, calming or stress reduction techniques”, and “learning to be aware of automatic thoughts or reactions” (Table 3).

### 3.5 Side Effects of Opioids

Most participants (68.0%) reported experiencing “side effects or other problems related to opioid pain medications,” with the majority noting the interventions helped them cope with them (Table 2). The most frequently reported opioid therapy‐related problems were “constipation or digestive issues” (8 CBT, 7 MBT), “drowsiness or fatigue” (5 CBT, 7 MBT), and “impaired cognition” (4 CBT, 7 MBT).

When asked how the interventions helped cope, the most frequently identified strategies included “learning to be aware of automatic thoughts or beliefs or reactions”, “learning to practice meditation or present‐moment awareness”, and “training motivated me to reduce opioid medications” (Table 3).

### 3.6 Intervention Safety

Questions about the intervention's negative effects were accidentally omitted during 7 interviews; thus, 43 participants (22 MBT, 21 CBT) provided responses. A small number of these participants (3 MBT, 2 CBT) reported mild, transient, intervention‐related negative effects, which were generally brief and occurred mainly during a specific practice. One MBT participant felt distressed by a mental image during the “walking down the street” practice; another recalled past trauma during the “challenging pain situations” exercise; and a third experienced worsened pain during the “mindfulness of pain” exercise. One CBT participant felt distress upon learning the pain would likely persist, and another reported muscle cramps during the progressive muscle relaxation practice.

## 4 Discussion

This qualitative study, conducted as part of a large RCT of MBT versus CBT for adults with opioid‐treated CLBP [23, 24], aimed to understand whether and how the STAMP interventions supported improvements in daily function, pain, QoL, and opioid use. Through in‐depth exit interviews, the qualitative data provided critical context to the quantitative outcomes (published elsewhere) [16] and amplified participant voices, offering insights into the lived experiences of adults with opioid‐treated CLBP behind the numerical findings.

Our results provide possible explanation on how the MBT and CBT interventions may exert benefits and corroborate prior evidence on the effectiveness and safety of MBT and CBT in chronic pain management, including CLBP [2, 12, 38, 39]. Cherkin et al.'s trial demonstrated that MBT and CBT led to greater improvements in back pain and functional limitations compared to usual care, with no significant differences between the two interventions, highlighting MBT as a viable therapeutic alternative in CLBP [2]. Cattanach et al. conducted a qualitative analysis of patient experiences with cognitive therapy, mindfulness meditation, and mindfulness‐based cognitive therapy for CLBP [12]. Participants identified both unique and shared therapeutic techniques, such as cognitive restructuring, present‐moment awareness, and coping skill development, as particularly beneficial. Common factors like therapeutic rapport and emotional, cognitive, and behavioral changes were also key to positive outcomes. That study supported the overall efficacy and acceptability of these treatments for managing CLBP [12]. Horton et al. reported that a majority of the STAMP study participants (81.5%) across both MBT and CBT arms who provided brief qualitative responses expressed positive reflections on their intervention experience, citing benefits in pain management (31.5%), mindfulness practices (25.9%), and relaxation techniques (22.2%) [25]. While this analysis was based on brief qualitative assessments of all STAMP participants, our findings, from a subgroup of 50 participants who reported improvements, build upon and complement Horton et al.'s results.

Participants in our qualitative study frequently attributed improvements to the STAMP interventions, with an overall similar distribution across the MBT and CBT groups of those reporting benefits for pain, functional ability, QoL, opioid use reduction, and better coping with adverse effects of opioids. Many described relief not only from physical symptoms but also from emotional distress linked to opioid‐treated CLBP. Additionally, although most participants reported adverse effects from opioid medications, particularly constipation, fatigue, and cognitive impairment, they noted the study intervention‐based strategies helped them manage these negative effects. Techniques learned through the MBT or CBT training were cited by the majority of participants, across both groups, as “most helpful” for back pain care, with the most common ones including “learning to engage in physical activity or keeping active,” “learning relaxation, calming or stress reduction techniques,” “learning to be aware of automatic thoughts or beliefs or reactions,” and “learning to practice meditation or present‐moment awareness.” In addition, direct inquiry among the study participants about any adverse effects attributable to the study interventions indicated that MBT and CBT were safe. Overall, these results suggest that both MBT and CBT can exert lasting benefits in refractory, complex CLBP through both unique and shared therapeutic techniques. It seems likely the benefits of various non‐pharmacologic treatments are conferred by numerous mechanisms, many of them shared across individual interventions. Increasing physical activity and exercise, reducing pain‐amplifying negative cognitions (e.g., catastrophizing, fear of pain), developing skills to reduce the intensity or impact of daily pain symptoms, enhancing self‐efficacy for pain self‐management, improving sleep quality, and numerous other potential mechanistic processes have all been shown to directly impact pain symptomatology; while identifying neurophysiological pathways is beyond the scope of the present report, interventions such as MBT and CBT have been shown to adaptively alter the central nervous system's processing of pain‐related information, which may represent a “final common pathway” for non‐pharmacologic pain treatments [41].

These qualitative findings reinforced and contextualized the quantitative outcomes of the larger, parent STAMP trial, suggesting that both MBT and CBT are safe and effective in improving health and QoL, while also facilitating opioid dose reduction among a substantial subset of interviewed participants. Thus, this study highlights the potential of MBT and CBT to address complex, refractory pain and reduce opioid‐related harms. Despite evidence indicating their safety and effectiveness, these interventions are in general not widely accessible or covered by insurance. Increased recognition of MBT and CBT as evidence‐based treatments by clinicians, patients, and payers is warranted.

Several limitations should be acknowledged. The sample was not demographically representative; it included predominantly white, non‐Hispanic females with higher education, which may limit generalizability. Qualitative data, by nature, rely on self‐report and subjective interpretation, which may be influenced by recall bias or social desirability; we aimed to reduce the potential bias by employing a separate team of researchers who had not been previously involved with the study participants. Inclusion was restricted to participants who attended the MBT or CBT sessions and reported satisfaction with the study interventions and improvements in their numerically‐measured outcomes at 12 months, compared to baseline, introducing selection bias and underrepresenting potential negative or neutral experiences. The absence of a usual care‐only comparison group precluded drawing firm conclusions about the effectiveness of the study interventions; however, the existing research evidence, experiences of our stakeholder‐partners, and participant qualitative reports [25] suggest that improvements in both the MBT and CBT groups were associated with the study interventions.

Future research should focus on optimizing intervention content and delivery, for example by identifying the most effective components of MBT and CBT (e.g., such as those qualitatively reported by STAMP participants in this study), enabling shortening of the interventions as the full programs are resource‐intensive and may limit scalability. Tailoring these interventions to individual needs and improving accessibility will be essential for broader implementation.

## 5 Conclusions

MBT and CBT can be safe and effective in reducing symptoms and opioid use in adults with opioid‐treated CLBP. The qualitative data provided participant‐centered perspectives that enriched understanding of the interventions' real‐world impact among those who benefited from the MBT or CBT interventions. Our findings underscore the need for broader access to MBT and CBT as essential components of evidence‐based, personalized care for individuals facing the challenges of refractory chronic pain treated with long‐term opioids.

## Author Contributions

Conceptualization: Eric L. Garland, Robert R. Edwards, Bruce Barrett, Aleksandra E. Zgierska. Data curation: Eric L. Garland, Robert R. Edwards, Cindy A. Burzinski, Kenneth D. Croes, Robert M. Cradock, Aleksandra E. Zgierska. Formal analysis: Eric L. Garland, Robert R. Edwards, Bruce Barrett, Cindy A. Burzinski, Kenneth D. Croes, Robert M. Cradock, Chan Shen, Aleksandra E. Zgierska. Funding acquisition: Eric L. Garland, Robert R. Edwards, Bruce Barrett, Aleksandra E. Zgierska. Investigation: Douwné L. Müller, Robert R. Edwards, Bruce Barrett, Kenneth D. Croes, Robert M. Cradock, Chan Shen, Shilagh A. Mirgain, Singles, Aleksandra E. Zgierska. Methodology: Eric L. Garland, Robert R. Edwards, Bruce Barrett, Robert M. Cradock, Aleksandra E. Zgierska. Project administration: Eric L. Garland, Robert R. Edwards, Bruce Barrett, Cindy A. Burzinski, Kenneth D. Croes, Shilagh A. Mirgain, Janice M. Singles, Aleksandra E. Zgierska. Resources: Eric L. Garland, Robert R. Edwards, Bruce Barrett, Aleksandra E. Zgierska. Software: Kenneth D. Croes, Robert M. Cradock, Chan Shen. Supervision: Eric L. Garland, Robert R. Edwards, Bruce Barrett, Aleksandra E. Zgierska. Validation: Eric L. Garland, Robert R. Edwards, Cindy A. Burzinski, Kenneth D. Croes, Aleksandra E. Zgierska. Visualization: Douwné L. Müller, Aleksandra E. Zgierska. Writing – original draft: Douwné L. Müller, Kenneth D. Croes, Chan Shen, Aleksandra E. Zgierska. Writing – review and editing: Douwné L. Müller, Eric L. Garland, Robert R. Edwards, Bruce Barrett, Cindy A. Burzinski, Kenneth D. Croes, Robert M. Cradock, Chan Shen, Shilagh A. Mirgain, Janice M. Singles, Aleksandra E. Zgierska.

## Funding

This work was supported by the Patient‐Centered Outcomes Research Institute (PCORI) award (OPD‐1601‐33860). The views and statements in this publication are solely the responsibility of the authors and do not necessarily represent the views of the PCORI, its Board of Governors or Methodology Committee. This project also received funding and institutional support through the University of Wisconsin‐Madison School of Medicine and Public Health, and its Department of Family Medicine and Community Health; Brigham and Women's Hospital, Harvard Medical School; University of Utah College of Social Work; and The Pennsylvania State University Department of Family and Community Medicine.

## Ethics Statement

This study was approved by the Institutional Review Boards (IRBs) of all participating institutions, with the University of Wisconsin–Madison's IRB serving as the IRB of record (study #2017–0353). A Certificate of Confidentiality was issued by the National Institutes of Health to protect participant privacy. The trial was registered with ClinicalTrials.gov (NCT03115359) prior to participant enrollment. An independent Data and Safety Monitoring Committee reviewed study progress and participant safety biannually.

## Consent

All participants provided written informed consent prior to enrollment in the study.

## Conflicts of Interest

Dr. Zgierska reported serving on the board of directors and executive committee for the American Society of Addiction Medicine and receiving reimbursement of some of the travel cost 1–2 times per year to in‐person meetings of this society outside the submitted work. Dr. Garland reported serving as the Director of UCSD ONEMIND (Optimized Neuroscience‐Enhanced Mindfulness Intervention Design), which provides mindfulness‐oriented recovery enhancement (MORE), mindfulness‐based therapy, and cognitive behavioral therapy in the context of research trials for no cost to research participants; receiving honoraria and payment for delivering seminars, lectures, and teaching engagements (related to training clinicians in mindfulness), including those sponsored by institutions of higher education, government agencies, academic teaching hospitals, and medical centers; receiving royalties from the sale of books related to MORE; and being a licensor to BehaVR LLC. The other authors declare no conflicts of interest.

## Supporting information


**Table S1:** Additional baseline characteristics of the study sample.
Supplement 1. Guide for the in‐depth interviews.
**Table S2:** Change in primary and secondary outcomes and participant‐perceived change for “better” at 12 months compared to baseline.

## Data Availability

Quantitative data for the present study can be accessed through the Patient‐Centered Outcomes Research Institute (PCORI)'s registry (when it becomes available). Due to institutional policies and the sensitive nature of qualitative data, they may be shared upon reasonable request, following completion of data sharing use agreements.
