# Supplementary material for: How Do Mindfulness‐Based and Cognitive Behavioral Therapies Help Adults With Opioid‐Treated Chronic Low Back Pain? A Qualitative Study
Source: Pain Pract. 2026 Oct 9;26(8):e70209. doi: 10.1111/papr.70209 (PMC13653131; doi:10.1111/papr.70209)
Supplement: Supplementary file 1 [file PAPR-26-0-s001.docx]

**Supplementary Material**

Supplement 1. Guide for the In-Depth Interviews.

Supplemental Table 1. Additional baseline characteristics of the study sample.

Supplemental Table 2. Change in primary and secondary outcomes and participant-perceived change for “better” at 12 months compared to baseline.

**Supplement 1. Guide for the In-Depth Interviews.**

Abbreviations: CBT: Cognitive Behavioral Therapy; MBT: Mindfulness-Based Therapy; R: respondent (i.e., the interviewed participant); STAMP: Strategies To Assist with Management of Pain study

**INTRODUCTION**

Hello, my name is ______ [INTERVIEWER NAME]. I’m a researcher with the pain management study that you have been participating in over the last year, the STAMP study. I believe that ______ [SITE-SPECIFIC CONTACT NAME] let you know that I would be calling, is that right?

(As ___ [SITE-SPECIFIC CONTACT NAME] may have mentioned,) We are reaching out to you and other study participants to learn whether the training you received has affected your back pain and use of back pain medications. Your input looking back at your experiences over the past 12 months would be very helpful.

I have several questions for us to talk through. Would now be a good time?

INTERVIEWER: IF R ASKS, THE INTERVIEW WILL TAKE 30-45 MINUTES. IT CAN BE COMPLETED IN MORE THAN ONE TELEPHONE CALL.

IF NO, SCHEDULE A DIFFERENT TIME.

IF YES: Your participation in this interview is completely voluntary. I’d like to audio record so that I don’t miss any of your comments. I’ll use the recording to make notes for a report. I may quote you in the report, but I will not use your name or the names of other study participants. The recordings will be kept private and secure, and only the research team will be able to hear them. May I have your permission to turn on the recorder?

IF NO: THANK R, TERMINATE THE INTERVIEW.

IF YES: INTERVIEWER, START THE RECORDER NOW. Okay, thank you, the recorder is on now. Please let me know if you would like me to pause the recorder or turn it off at any time.

**SECTION 1. TRAINING RECEIVED**

BASED ON THE STUDY RECORD, R RECEIVED:

__ MBT

__ CBT

100.	Today we’ll be talking about the effects of the training you received about a year ago as part of this study to help you manage your back pain.

101.	One group of participants received training in mindfulness meditation. And the other group was trained in cognitive behavioral therapy. Which training did you receive?

FOLLOW-UP: How should I refer to the training you received?

INTERVIEWER: SUBSTITUTE R’S PREFERRED TERM FOR “MBT/CBT” BELOW.

INTERVIEWER: IF R WAS ASSIGNED TO ONE TYPE OF TRAINING BUT TALKS ABOUT THE OTHER TYPE OF TRAINING:

- ASK ABOUT R’s BACKGROUND WITH/TRAINING IN THE UNASSIGNED TRAINING,
- LET R KNOW THE INTERVIEW FOCUSES ON THE ASSIGNED TRAINING AND THAT THEY SHOULD RESPOND TO THE BEST OF THEIR ABILITY ABOUT THE TRAINING THEY RECEIVED AS PART OF STAMP.
- IF NOMENCLATURE BECOMES CONFUSING, REFER TO THE TRAINING AS “THE STUDY-PROVIDED TRAINING” THUS AVOIDING “CBT” AND “MBT” ALTOGETHER.

**SECTION 2. BACK PAIN**

200.	My first questions are about your back pain.

201.	[IF “AVERAGE PAIN SCORE (DIFFERENCE)” IN THE “BASELINE EXIT TABLE” IS RED (I.E., WORSENING FROM BASELINE), GO TO 202] Over the past 12 months or so, you filled out surveys about your back pain. Comparing those surveys over time, it looks like your back pain has gone down over the last year, is that correct?

__YES 🡪 GO TO 203

__NO 🡪 GO TO 202

202.	Over the past 12 months or so, has anything at all helped you reduce your back pain?

__YES 🡪 GO TO 203

__NO 🡪 GO TO 206

203.	What has helped you reduce your back pain?

INTERVIEWER: R MAY TALK ABOUT PAIN MEDICATION.

INTERVIEWER: MAKE A LIST OF WHAT HAS HELPED R REDUCE BACK PAIN IN THE EVENT THAT, BELOW, R SAYS “EVERYTHING I SAID BEFORE.”

204.	Is there anything else that has helped you reduce your back pain?

__YES 🡪 GO TO 205

__NO 🡪 GO TO 206

205.	What else (has helped you reduce your back pain)?

__ANYTHING OTHER THAN MBT/CBT 🡪 GO TO 206

__MBT/CBT 🡪 GO TO 207

206.	Has MBT/CBT helped you reduce your back pain in any way?

__YES 🡪 GO TO 207

__NO 🡪 GO TO 208

207.	How (has MBT/CBT helped you reduce your back pain)?

PROBE: How else has MBT/CBT helped you reduce your back pain?

208.	When you think of all the things you’ve done to reduce your back pain over the past 12 months – whether it’s part of the MBT/CBT training or other things – what has been the most helpful?

209	Have you ever experienced any mood problems, stress, or anxiety about your back pain?

__YES 🡪 GO TO 210

__NO 🡪 GO TO 212

210.	Has MBT/CBT helped you improve your mood, stress, or anxiety about your back pain in any way?

__YES 🡪 GO TO 211

__NO 🡪 GO TO 212

IF R TALKS ABOUT MBT/CBT’s EFFECTS ON BACK PAIN, PROBE: This question is asking if MBT/CBT has helped you improve your mood, stress, or anxiety about your back pain.

211.	How (has MBT/CBT helped you improve your mood, stress, or anxiety about your back pain?)

PROBE: How has MBT/CBT helped you improve the way you think or feel about your back pain?

212.	What advice would you give others in your situation to help them reduce their back pain?

**SECTION 3. FUNCTIONING**

300.	My next questions are about your ability to do your daily activities. Daily activities are the things we normally do, such as bathing, dressing, walking, preparing meals, taking care of yourself or your household, or interacting with others. These are just a few examples; there are many other types of daily activity. Please think about your own daily activities for the following questions.

301.	[IF “PAIN INTERFERENCE SCORE (DIFFERENCE)” IN THE “BASELINE EXIT TABLE” IS RED (I.E., WORSENING FROM BASELINE) GO TO 302] Comparing, again, the surveys you filled out over the past 12 months or so, it looks like your ability to do at least some of your daily activities has improved, is that correct?

__YES 🡪 GO TO 303

__NO 🡪 GO TO 302

302.	Over the past 12 months or so, has anything at all helped you improve your ability to do at least some of your daily activities?

__YES 🡪 GO TO 303

__NO 🡪 GO TO 307

PROMPT IF CLARIFICATION IS NEEDED: For example, physical activity of any specific type; icing; rest.

303.	What daily activities have improved for you?

304.	What has helped you improve your ability to do your daily activities?

305.	Is there anything else that has helped you improve your ability to do your daily activities?

__YES 🡪 GO TO 306

__NO 🡪 GO TO 307

306.	What else (has helped you improve your ability to do your daily activities)?

__ANYTHING OTHER THAN MBT/CBT 🡪 GO TO 307

__MBT/CBT 🡪 GO TO 308

307.	Has MBT/CBT helped you improve your ability to do your daily activities in any way?

__YES 🡪 GO TO 308

__NO 🡪 GO TO 309

308.	How (has MBT/CBT helped you improve your ability to do your daily activities)?

PROBE: How else has MBT/CBT helped you improve your ability to do your daily activities?

309.	When you think of all the things that have helped you improve your ability to do your daily activities over the past 12 months – whether it’s part of the MBT/CBT training or other things – what has been the most helpful?

310.	Have you ever experienced any mood problems, stress, or anxiety about your ability to do your daily activities?

__YES 🡪 GO TO 311

__NO 🡪 GO TO 313

311.	Has MBT/CBT helped you improve your mood, stress, or anxiety about your ability to do your daily activities in any way?

__YES 🡪 GO TO 312

__NO 🡪 GO TO 313

INTERVIEWER: IF R TALKS ABOUT MBT/CBT’s EFFECTS ON DAILY ACTIVITIES, PROBE: This question is asking if MBT/CBT has helped you improve your mood, stress, or anxiety about your ability to do your daily activities.

312.	How (has MBT/CBT helped you improve your mood, stress, or anxiety about your ability to do your daily activities?)

PROBE: How has MBT/CBT helped you improve the way you think or feel about your ability to do your daily activities?

313.	What advice would you give others in your situation to help them improve their ability to do daily activities?

**SECTION 4. OPIOID MEDICATIONS**

400.	My next questions are about pain medication.

401.	[IF “MORPHINE-EQUIVALENT DOSE, MG/DAY (DIFFERENCE)” IN THE “BASELINE EXIT TABLE” IS RED (I.E., INCREASED FROM BASELINE), GO TO 402] Comparing, once again, the surveys you filled out over the past 12 months, it looks like your dosage of opioid medications to treat your back pain has gone down over the last year, is that correct?

__YES 🡪 GO TO 403

__NO 🡪 GO TO 402

402.	Over the past 12 months or so, has your dosage of opioid medications to treat your back pain gone down at all?

__YES 🡪 GO TO 403

__NO 🡪 GO TO 406

403.	What has helped you reduce your use of opioid medications for your back pain?

404.	Is there anything else that has helped you reduce your use of opioid medications for your back pain?

__YES 🡪 GO TO 405

__NO 🡪 GO TO 406

405.	What else (has helped you reduce your use of opioid medications for your back pain)?

__ANYTHING OTHER THAN MBT/CBT 🡪 GO TO 406

__MBT/CBT 🡪 GO TO 407

406.	Has MBT/CBT helped you reduce your use of opioid medications for your back pain in any way?

__YES 🡪 GO TO 407

__NO 🡪 GO TO 408

407.	How (has MBT/CBT helped you reduce your use of opioid medications for your back pain)?

PROBE: How else has MBT/CBT helped you reduce your use of opioid medications for your back pain?

408.	When you think of all the things you’ve done to reduce your use of opioid medications for your back pain over the past 12 months – whether it’s the MBT/CBT training or other things – what has been the most helpful?

409.	Have you ever experienced any mood problems, stress, or anxiety about your use of opioid medications?

__YES 🡪 GO TO 410

__NO 🡪 GO TO 412

410.	Has MBT/CBT helped you improve your mood, stress, or anxiety about your use of opioid medications in any way?

__YES 🡪 GO TO 411

__NO 🡪 GO TO 412

411.	How (has MBT/CBT helped you improve your mood, stress, or anxiety about your use of opioid medications)?

PROBE: How has MBT/CBT helped you improve the way you think or feel about your use of opioid medications?

412.	What advice would you give others in your situation to help them reduce their use of opioid medications for back pain?

**SECTION 5. SIDE EFFECTS**

500.	My next questions are about side effects.

**OPIOID MEDICATIONS**

501.	Have you ever experienced side effects or other problems related to your opioid pain medications?

__YES 🡪 GO TO 502

__NO 🡪 GO TO 505

INTERVIEWER: IF R NEEDS A DEFINITION OF “SIDE EFFECTS”:

- A SIDE EFFECT WOULD BE ANYTHING THAT IS MADE WORSE BY TAKING OPIOIDS.
- IF PARTICIPANTS BRING UP SOCIAL RAMIFICATIONS OF OPIOIDS (E.G., STIGMA, WORSE RELATIONSHIP WITH PROVIDERS, ETC., IT IS VALID AS WELL).

502.	Can you briefly describe them (the side effects or other problems that you have experienced related to your opioid medication)?

503.	Has MBT/CBT helped you in any way to cope with any of these side effects or other problems?

__YES 🡪 GO TO 504

__NO 🡪 GO TO 505

504.	How (has MBT/CBT helped you to cope with these side effects or other problems?

**STUDY INTERVENTIONS**

505.	Have you experienced any negative effects as a result of practicing MBT/CBT?

__YES 🡪 GO TO 506

__NO 🡪 GO TO 600

PROBE: Did anything get worse for you due to MBT/CBT?

506.	What negative effects have you experienced as a result of practicing MBT/CBT?

**SECTION 6: FINAL QUESTIONS**

600.	That brings us to the end of our questions.

601.	Is there anything you’d like to add to what you have said already?

**Supplemental Table 1. Additional baseline characteristics of the study sample.**

Approximately one-half of the study participants (N=50) reported obtaining a college degree. The majority of participants reported a history of being separated, divorced or widowed (58.0%), and being now in a relationship (60.0%). Only 20.0% of participants endorsed currently working, and 44.0% reported a gross individual income below $25,000. The majority had experienced back pain for more than five years (70.0%) and had tried various prior treatments for CLBP. Approximately one-half of participants believed their back pain was terrible and not going to get better, while 34.0% felt that being physically active was not safe for someone with their condition.

|  | Total Sample  *N=50* | MBT Group  *N=26* | CBT Group  *N=24* |
| --- | --- | --- | --- |
| Demographic Characteristics Continued | | | |
| Education, n (%): | | | |
| No high school diploma | 2 (4.0%) | 1 (3.9%) | 1 (4.2%) |
| High school graduate or GED | 3 (6.0%) | 2 (7.7%) | 1 (4.2%) |
| Some college, no degree | 9 (18.0%) | 5 (19.2%) | 4 (16.7%) |
| Occupational/technical/vocational program | 4 (8.0%) | 3 (11.5%) | 1 (4.2%) |
| Associate degree: academic program | 7 (14.0%) | 4 (15.4%) | 3 (12.5%) |
| Bachelor’s degree | 12 (24.0%) | 5 (19.2%) | 7 (29.2%) |
| Master’s degree | 11 (22.0%) | 5 (19.2%) | 6 (25.0%) |
| Doctoral degree | 2 (4.0%) | 1 (3.9%) | 1 (4.2%) |
| Current Relationship Status n (%): | | | |
| Single, not in a relation | 20 (40.0%) | 9 (34.6%) | 11 (45.8%) |
| In a relationship, or Married | 30 (60.0%) | 17 (65.4%) | 13 (54.2%) |
| Past relationship/married status n (%): | | | |
| Divorced/Separated/Widowed | 29 (58.0%) | 16 (61.5%) | 13 (54.2%) |
| Neither | 19 (38.0%) | 10 (38.5%) | 9 (37.5%) |
| Gross individual income n (%): | | | |
| Under $16,000 | 14 (28.0%) | 7 (26.9%) | 7 (29.2%) |
| $16,000-$24,999 | 8 (16.0%) | 5 (19.2%) | 3 (12.5%) |
| $25,000-$34,999 | 8 (16.0%) | 2 (7.7%) | 6 (25.0%) |
| $35,000-$49,999 | 4 (8.0%) | 1 (3.9%) | 3 (12.5%) |
| $50,000-$74,999 | 4 (8.0%) | 4 (15.4%) | 0 (0.0%) |
| $75,000-$99,999 | 4 (8.0%) | 1 (3.9%) | 3 (12.5%) |
| $100,000 and above | 6 (12.0%) | 4 (15.4%) | 2 (8.3%) |
| Employment n (%): | | | |
| Working now | 10 (20.0%) | 6 (23.1%) | 4 (16.7%) |
| Unemployed | 3 (6.0%) | 2 (7.7%) | 1 (4.2%) |
| Disabled (any reason) | 25 (50.0%) | 12 (46.2%) | 13 (54.2%) |
| Retired | 9 (18.0%) | 5 (19.2%) | 4 (16.7%) |
| Other | 3 (6.0%) | 1 (3.9%) | 2 (8.3%) |
| Chronic Low Back Pain-Related Characteristics (NIH MD) | | | |
| CLBP duration, n (%): | | | |
| 6 months–1 year | 2 (4.0%) | 2 (7.7%) | 0 (0.0%) |
| 1–5 years | 13 (26.0%) | 8 (30.8%) | 5 (20.8%) |
| > 5 years | 35 (70.0%) | 16 (61.5%) | 19 (79.2%) |
| Back pain spreading down the leg(s), past 2 weeks, n (%): | | | |
| Yes | 39 (78.0%) | 20 (76.9%) | 19 (79.2%) |
| No | 9 (18.0%) | 5 (19.2%) | 4 (16.7%) |
| Off work or unemployed for ≥1 month due to low back pain, n (%): | | | |
| Yes | 21 (42.0%) | 11 (42.3%) | 10 (41.7%) |
| No | 12 (24.0%) | 6 (23.1%) | 6 (25.0%) |
| Receiving/applied for disability or workers’ compensation due to low back pain, n (%): | | | |
| Yes | 21 (42.0%) | 9 (34.6%) | 12 (50.0%) |
| No | 18 (36.0%) | 10 (38.5%) | 8 (33.3%) |
| I feel my back pain is terrible and never going to get any better, n (%): | | | |
| Yes | 26 (52.0%) | 10 (38.5%) | 16 (66.7%) |
| No | 21 (42.0%) | 15 (57.7%) | 6 (25.0%) |
| It’s not really safe for a person with my back problem to be physically active, n (%): | | | |
| Yes | 17 (34.0%) | 7 (26.9%) | 10 (41.7%) |
| No | 32 (64.0%) | 18 (69.2%) | 14 (58.3%) |
| Prior treatments for back pain | | | |
| % Relief from opioid medications,  past 7 days, mean (SD) | 59.0 (21.2) | 66.5 (17.2) | 50.8 (22.4) |
| History of a low back operation, n (%)  Yes, one or more operations  No operation | 23 (46.0%)  27 (54.0%) | 13 (50.0%)  13 (50.0%) | 10 (41.7%)  14 (58.3%) |
| Injections (e.g., epidural steroid, facet), n (%)  Yes  No | 41 (82.0%)  7 (14.0%) | 21 (80.8%)  3 (11.5%) | 20 (83.3%)  4 (16.7%) |
| Exercise therapy (physical therapy), n (%)  Yes  No | 47 (94.0%)  2 (4.0%) | 25 (96.2%)  0 (0.0%) | 22 (91.7%)  2 (8.3%) |
| Psychological counseling, n (%)  Yes  No | 18 (36.0%)  30 (60.0%) | 7 (26.9%)  17 (65.4%) | 11 (45.8%)  13 (54.2%) |
| Healthcare Utilization and Productivity Characteristics | | | |
| Hospitalized, n (%): | | | |
| Yes | 12 (24.5%) | 8 (32.0%) | 4 (16.7%) |
| No | 37 (75.5%) | 17 (68.0%) | 20 (83.3%) |
| The number of nights hospitalized, mean (SD) | 1.1 (3.2) | 1.8 (4.3) | 0.4 (1.0) |
| Emergency Department visit, n (%): | | | |
| Yes | 16 (33.3%) | 10 (41.7%) | 6 (25.0%) |
| No | 32 (66.7%) | 14 (58.3%) | 18 (75.0%) |
| The number of ED visit, mean (SD) | 0.8 (1.9) | 0.6 (0.9) | 0.9 (2.5) |
| Clinic visit (regular/urgent care) n (%): | | | |
| Yes | 45 (90.0%) | 24 (92.3%) | 21 (87.5%) |
| No | 5 (10.0%) | 2 (7.7%) | 3 (12.5%) |
| The number of clinic visits, mean (SD) | 4.5 (4.5) | 5.4 (5.3) | 3.6 (3.4) |
| Mental health care visits, n (%): | | | |
| Yes | 20 (40.0%) | 10 (38.5%) | 10 (41.7%) |
| No | 30 (60.0%) | 16 (61.5%) | 14 (58.3%) |
| The number of Mental health care visits, mean (SD) | 3.1 (6.5) | 2.0 (4.2) | 4.3 (8.2) |
| Chores (school/house) missed, n (%): | | | |
| Yes | 32 (71.1%) | 19 (79.2%) | 13 (61.9%) |
| No | 13 (28.9%) | 5 (20.8%) | 8 (38.1%) |
| The number of whole days of chores missed, mean (SD) | 27.6 (48.3) | 29.0 (52.0) | 25.9 (45.1) |
| Whole workdays missed (among those working now), n (%): | | | |
| Yes | 6 (60.0%) | 5 (83.3%) | 1 (25.0%) |
| No | 4 (40.0%) | 1 (16.7%) | 3 (75.0%) |
| The number of whole workdays missed, mean (SD) | 17.9 (49.4) | 6.5 (9.7) | 28.8 (67.3) |

The frequencies/percentages in a given variable category may not total the sample size per group or 100.0% responses as some participants’ responses were unavailable (e.g., missing or marked as declined, unknown or unspecified).

*Acronyms:* CBT: Cognitive Behavioral Therapy; CLBP: Chronic Low Back Pain; MBT: Mindfulness-Based Therapy; NIH MD: National Institutes of Health Minimal Data Set for CLBP studies; SD: standard deviation

**Supplemental Table 2. Change in primary and secondary outcomes and participant-perceived change for “better” at 12 months compared to baseline within the MBT and CBT groups.**

| Variable | MBT Group  N=26 | | | CBT Group  N=24 | | |
| --- | --- | --- | --- | --- | --- | --- |
|  | **Baseline** | **12 Months** | **P value^1^** | **Baseline** | **12 Months** | **P value^1^** |
| Average pain severity (BPI, 0-10 score), mean (SD) | 5.4 (1.5) | 4.2 (1.5) | <0.001 | 6.1 (1.5) | 4.8 (2.1) | <0.001 |
| Functional limitations (ODI, 0-100 score), mean (SD) | 46.9 (13.7) | 31.3 (14.8) | <0.001 | 47.4 (13.9) | 34.7 (16.0) | <0.001 |
| Physical health-related QoL, (SF-12, 0-100 score), mean (SD) | 28.2 (8.6) | 34.9 (9.1) | <0.001 | 28.3 (8.8) | 33.9 (9.9) | <0.001 |
| Mental health-related QoL, (SF-12, 0-100 score), mean (SD) | 41.1 (10.9) | 48.6 (10.8) | <0.001 | 42.4 (10.8) | 49.6 (9.9) | 0.001 |
| Opioid dose (TLFB), log MME/day, mean (SD) | 5.3 (1.3) | 3.4 (2.3) | <0.001 | 5.5 (2.0) | 3.9 (3.3) | <0.001 |
| Participant-perceived change (PGIC) for “better^2^” at 12 months | | | | | | |
| Pain, n (%) | 19 (73.1%) | | | 17 (70.8%) | | |
| Functional limitations, n (%) | 18 (69.2%) | | | 16 (66.7%) | | |
| Need for opioid medication, n (%) | 20 (76.9%) | | | 14 (60.9%) | | |
| Overall health, n (%) | 22 (84.6%) | | | 19 (79.2%) | | |
| Overall treatment satisfaction, n (%) | 24 (92.3%) | | | 23 (95.8%) | | |

^1^ Paired-sample t-test assessing within group change

^2^ “Better” (responses “5-7”): change for better with a meaningful improvement

*Acronyms:* BPI: Brief Pain Inventory; CBT: Cognitive Behavioral Therapy; MBT: Mindfulness-Based Therapy; ODI: Oswestry Disability Index; PGIC: Patient Global Impression of Change; SD: standard deviation; SF-12: 12-Item Short Form Health Survey; TLFB: Timeline Followback method
